# Development of a multiplex probe combination-based one-step real-time reverse transcription-PCR for NA subtype typing of avian influenza virus

**DOI:** 10.1038/s41598-017-13768-4

**Published:** 2017-10-18

**Authors:** Zhihao Sun, Tao Qin, Feifei Meng, Sujuan Chen, Daxin Peng, Xiufan Liu

**Affiliations:** 1 College of Veterinary Medicine, Yangzhou University, Yangzhou, Jiangsu 225009 PR China; 2 Jiangsu Co-Innovation Center for the Prevention and Control of Important Animal Infectious Disease and Zoonoses, Yangzhou, Jiangsu 225009 PR China; 3 Jiangsu Research Centre of Engineering and Technology for Prevention and Control of Poultry Disease, Yangzhou, Jiangsu 225009 PR China

## Abstract

Nine influenza virus neuraminidase (NA) subtypes have been identified in poultry and wild birds. Few methods are available for rapid and simple NA subtyping. Here we developed a multiplex probe combination-based one-step real-time reverse transcriptase PCR (rRT-PCR) to detect nine avian influenza virus NA subtypes. Nine primer-probe pairs were assigned to three groups based on the different fluorescent dyes of the probes (FAM, HEX, or Texas Red). Each probe detected only one NA subtype, without cross reactivity. The detection limit was less than 100 EID_50_ or 100 copies of cDNA per reaction. Data obtained using this method with allantoic fluid samples isolated from live bird markets and H9N2-infected chickens correlated well with data obtained using virus isolation and sequencing, but was more sensitive. This new method provides a specific and sensitive alternative to conventional NA-subtyping methods.

## Introduction

Avian influenza viruses (AIVs) are divided into subtypes on the basis of two surface glycoproteins: hemagglutinin (HA) and neuraminidase (NA), which are antigenically divided into 16 HA (H1 to H16) and 9 NA (N1 to N9) subtypes. Each subtype is highly diverse genetically and has unique lineages that are partitioned geographically^1^. To date, all nine NA subtypes have been detected in aquatic birds, which serve as the primary reservoir of influenza A viruses^2^. One HA subtype can combine with different NA subtypes to form different reassortant viruses such as H5N1, H5N2, H5N5, H5N6, and H5N8^3–8^.

For NA subtyping, the traditional method is to perform neuraminidase inhibition (NI) assays with cultured AIVs, which is also the gold standard suggested by OIE (http://www.oie.int/fileadmin/Home/eng/Healthstandards/tahm/2.03.04_AI.pdf). However, the diversity of the reference antisera from different areas, cross reactivity, subjective evaluation, low sensitivity, low specificity, and low accuracy may affect the typing results^9^. Sequencing of NA genes is also used for subtyping, but this method is expensive, time-consuming and labor-intensive^10^.

PCR-based molecular diagnostic tests such as conventional reverse transcriptase PCR (RT-PCR)^11–14^ and real-time RT-PCR (rRT-PCR)^15,16^ are more efficient approaches for NA subtyping^17^. Most studies have focused on identifying partial NA subtypes, such as N1 and N2^18,19^. A few studies have detected all nine NA subtypes. For example, nine pairs of NA-specific RT-PCR primers were designed to amplify NA genes and the subtype of NA was determined by subsequent agarose gel electrophoresis^13^. A multiple PCR-based assays was developed and used to differentiate 9 AIV NA genes^20^. However, these methods were neither rapid nor especially sensitive.

Here we developed a rapid, sensitive, and specific multiplex probe combination-based one-step rRT-PCR for typing nine AIV NA subtypes. Nine primer-probe pairs were assigned into three groups based on the different fluorescent dyes of probes. The three groups reacted independently and no interference occurred within each group, which reduced the numbers of PCR reactions from 9 to 3, and increased detection specificity and sensitivity.

## Results

### Selection of primers and probes

**Figure 1:**
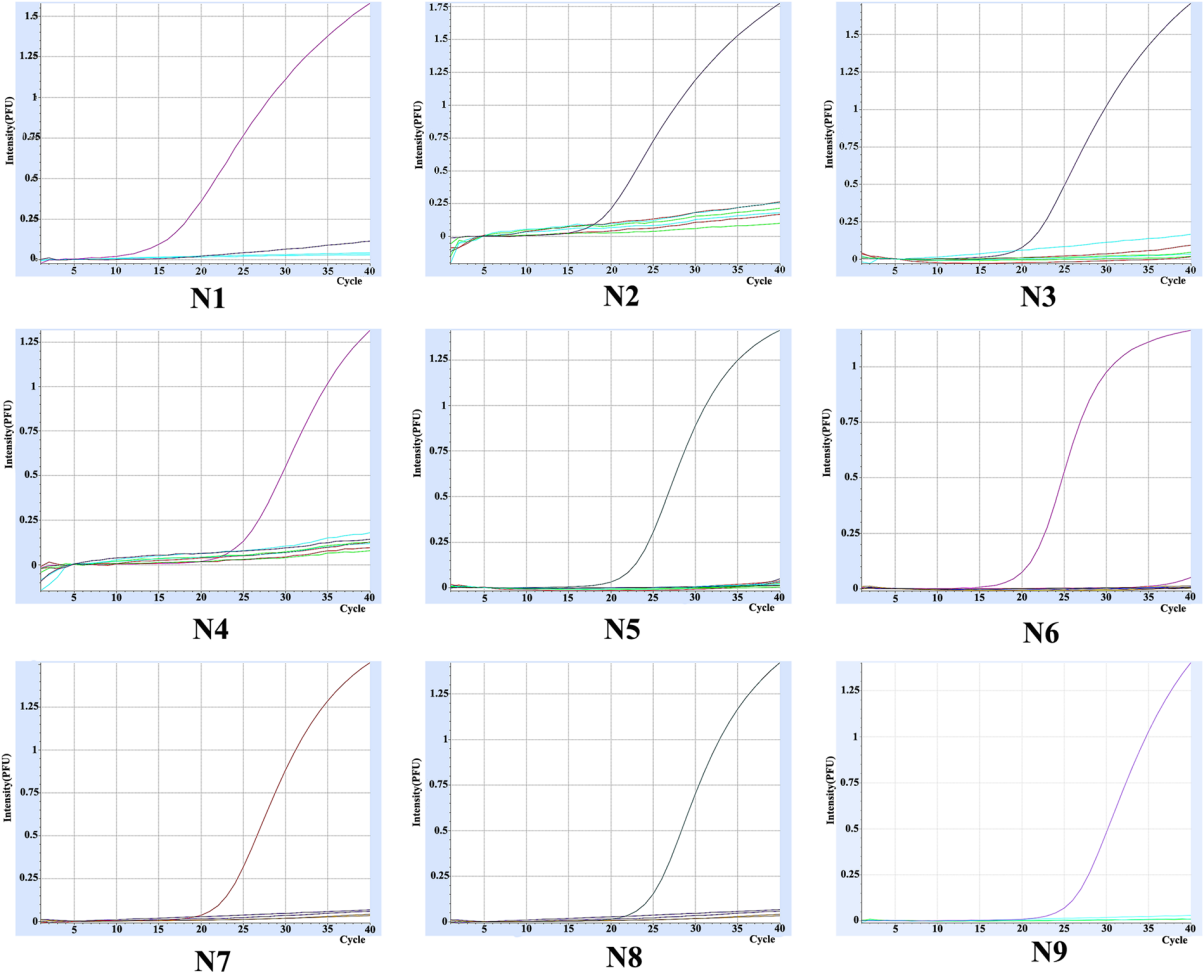
Nine amplification curves of corresponding NA subtypes in rRT-PCR. Total RNAs of nine different NA subtype avian influenza virus were extracted and used as templates for rRT-PCR.

**Figure 2:**
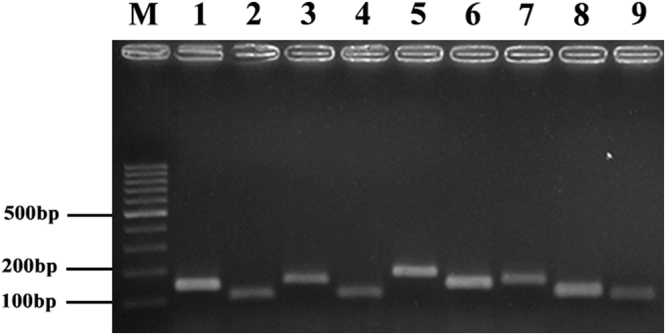
rRT-PCR product gel electrophoresis. Total RNAs of nine different NA subtype avian influenza virus were extracted and used as templates for rRT-PCR, rRT-PCR products were subjected to agarose gel electrophoresis and stained with ethidium bromide. Lanes 1–9, amplicons with primers specific to N1–N9 subtypes.

After visual inspection of the sequence alignments, 9 primer-probe pairs were designed (Table 1). Total RNAs of nine different NA subtype avian influenza virus were extracted and used as templates for rRT-PCR. Each primer-probe pair reacted with its corresponding NA subtype and appropriate amplification curves for each NA subtype were obtained (Fig. 1). Simultaneously, several other avian pathogens, including NDV, IBV, IBDV, adenovirus, and MDV were used as negative controls. There was no amplification of these templates using the nine primer-probe pairs in rRT-PCR. The PCR products were subjected to agarose gel electrophoresis and these sizes (105–187 bp) were as expected for each subtype (Fig. 2, Figure S1–1~S1–9).

**Table 1:** Primers, probes, and amplicon sizes of the rRT-PCR assays.

Group	NA subtype	Forward Primers (5′-3′)	Reverse Primers (5′-3′)	Probes (5′-3′)	Amplicon size(bp)
1	N1	TGTGTGTGCAGRGAYAAYTGSC	GGACCRCAACTSCCTGTHCCRTCV	FAM-CCACGCCCCAATGATGGAACAGGCAGTTG- BHQ1	137
	N4	ATGGTGTTTGGATAGGGAGGACAAA	CACCATTTGAATCCTTGTCTGTCGA	HEX-AGCTTGGAATCCAGAAGCGGTTTTGAGATGGT- BHQ1	105
	N5	AGAACACAAGAGTCTTCGTGTGTTT	TGGGATAACAGGAACATTCTTCAAT	TexasRed-AGAGTGTTATTGGGTAATGACGGACGGTCCA- BHQ1	178
2	N3	AGCAGTTGCTTCGATGGAAAG	CATTGACATTCAGACTCTTGAGTTC	FAM-ATGACCGGGAACGACAATGATGCGAGTGG- BHQ1	155
	N2	ACGAGTTGGGTGTCCCGTTTCATTT	TTCTATCATCCCCAGTGACACAAAC	HEX-TGGGAACCAAACAAGTGTGCATAGCATGGTC- BHQ1	117
	N6	AAGGGTGCAGGATGTTTGCTCTAAG	CCTAGTATTATATGGACTGGGTGCT	TexasRed-AAGGCACAACACTCAGAGGGCGACATGCAAAT- BHQ1	140
3	N7	ACTCAGGAGTCAGAATGTGTATGCC	TCAATATGTTTGGCTGATCCCTTTA	FAM-TGGCACATGTGCAGTTGTAATGACTGACGG- BHQ1	155
	N9	GCCCTGATAAGCTGGCCACT	TGCATTGTTGTTTGGTCCTGATATA	HEX-ATCACCGCCCACAGTGTACAACAGCAGGGT- BHQ1	117
	N8	AAGAAGTGGATGACGATTGGTGTAA	AGTCCTTAATATATCTCCTGCCCAG	TexasRed-CAGGGCCAGATTCTAAAGCAGTAGCAGTAG- BHQ2	138

Codes for mixed bases position: R = A/G, Y = C/T, S = G/C, H = A/C/T, V = A/G/C.

### The establishment of the combined multiple rRT-PCR

The primer-probe pairs were assigned into three groups based on the different fluorescent dyes of probes (FAM, HEX or Texas Red; Table 1). To assess the specificity of the combined multiplex rRT-PCR, the cross-reactivity of the primer-probe pairs was examined first using combined nine subtype NA plasmids at a concentration of 10^5^ copies/μl. All primer-probe pairs reacted only with their corresponding NA subtype with mean Ct values from 20.73 to 24.51 (Table 2), indicating that there was no interference among the primer-probe pairs in the multiplex assays. Further, 111 AIV isolates, whose NA subtypes were confirmed by sequencing or RT-PCR identification, were used to evaluate the specificity and coverage (Table 3). Each subtype could be identified using the multiplex assay (Table 4).

**Table 2:** Specificity analysis of the developed rRT-PCR using 9 NA plasmids.

**Group**	**NA Subtype**	NA plasmids(1 × 10^5^copies/PCR, Ct ± SD)								
		N1	N2	N3	N4	N5	N6	N7	N8	N9
1	N1	24.51 ± 0.30^a^	—	—	—	—	—	—	—	—
	N4	—^b^	—	—	23.36 ± 0.26	—	—	—	—	—
	N5	—	—	—	—	23.81 ± 0.09	—	—	—	—
2	N2	—	22.49 ± 0.19	—	—	—	—	—	—	—
	N3	—	—	23.58 ± 0.23	—	—	—	—	—	—
	N6	—	—	—	—	—	23.18 ± 0.23	—	—	—
3	N7	—	—	—	—	—	—	22.06 ± 0.10	—	—
	N8	—	—	—	—	—	—	—	20.73 ± 0.08	—
	N9	—	—	—	—	—	—	—	—	22.14 ± 0.61

^a^Ct value determined from three replicates;

^b^not detected.

**Table 3:** 111 AIV isolates confirmed by sequencing or RT-PCR identification.

**Group**	**NA Subtype**	**Source**	**Number of isolates**	**Total isolates**	**Homology**
1	N1	H5N1	8	20	90.3–99.9%
		H1N1	2		
		H3N1	10		
	N4	H8N4	2	2	85.8%
	N5	H6N5	2	2	79.4%
2	N2	H5N2	22	30	81.4–99.9%
		H9N2	8		
	N3	H10N3	5	5	99.5–99.8%
	N6	H4N6	3	20	87.7–99.7%
		H5N6	16		
		H11N6	1		
3	N7	H7N7	1	2	86.6%
		H10N7	1		
	N8	H5N8	20	30	87.0–99.9%
		H3N8	7		
		H6N8	3		
	N9	H7N9	19	20	89.3–99.9%
		H11N9	1

**Table 4:** Typing of avian influenza virus isolates using the developed rRT-PCR.

Group	Fluorescent dye								
	FAM			HEX			TexasRed		
1	N1	N4	N5	N1	N4	N5	N1	N4	N5
	+(20/20)^a^	—^b^	—	—	+(2/2)	—	—	—	+(2/2)
2	N3	N2	N6	N3	N2	N6	N3	N2	N6
	+(5/5)	—	—	—	+(30/30)	—	—	—	+(20/20)
3	N7	N9	N8	N7	N9	N8	N7	N9	N8
	+(2/2)	—	—	—	+(20/20)	—	—	—	+(30/30)

^a^Represents positive (No. of positive strains/No. of tested strains);

^b^not detected.

### Detection limit of the developed rRT-PCR

**Figure 3:**
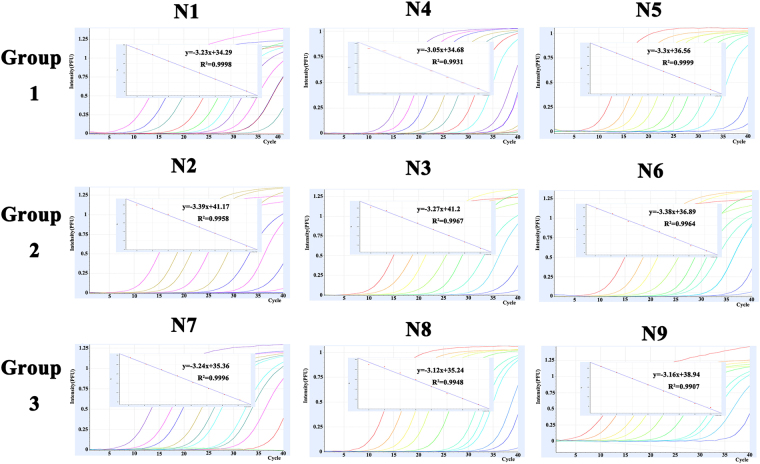
Amplification plots and standard curves of the multiplex assay. The multiplex assay was tested using nine NA plasmids ranging from 10^0^ to 10^9^ copies/μl. A PCR curve fit view of the data is shown with relative fluorescence units (RFUs) plotted against cycle numbers. Standard curves were generated from the Ct values obtained against known concentrations and the coefficient of determination (R^2^) and slope of the regression curve for each assay are indicated.

**Figure 4:**
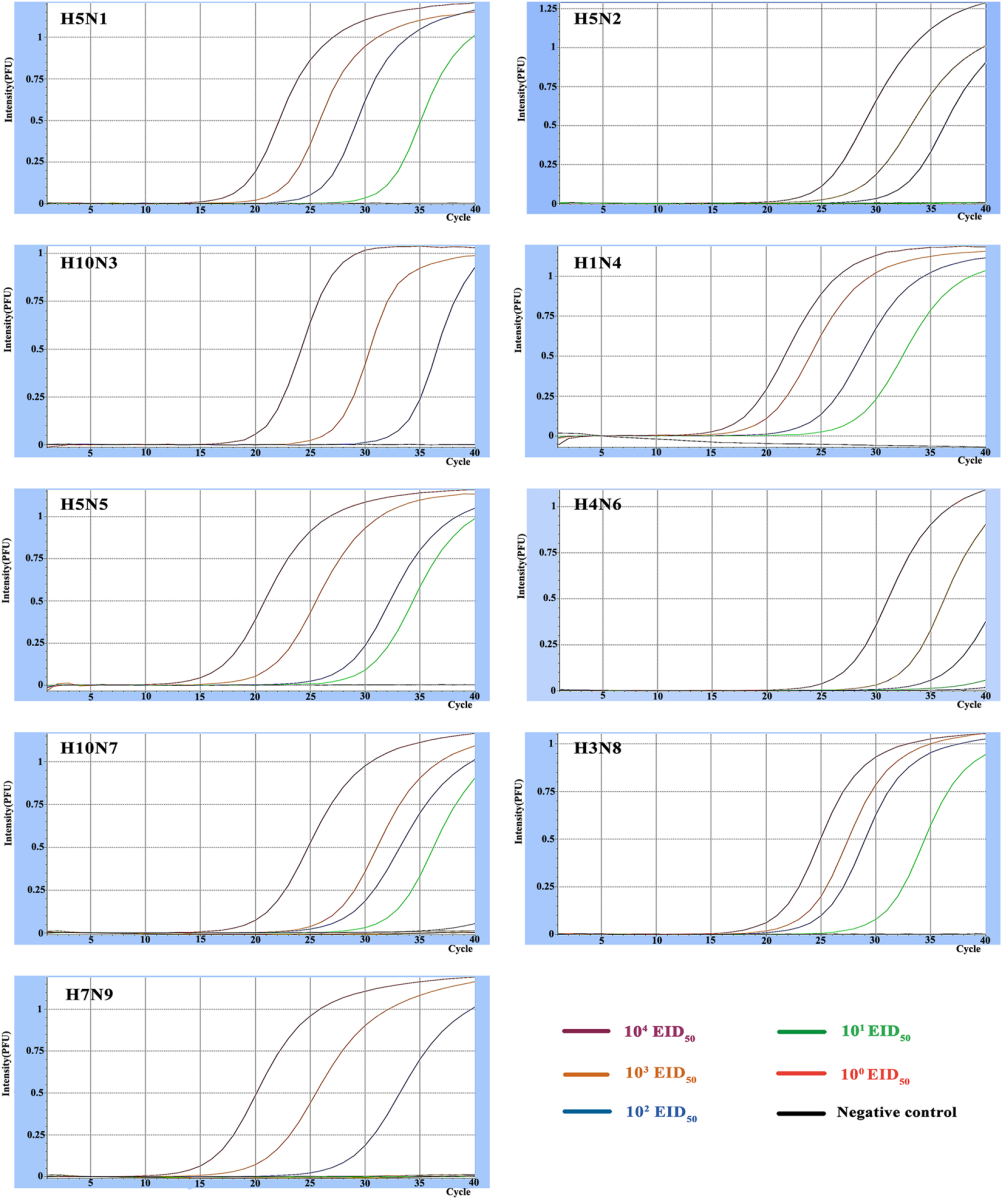
PCR detection limit of nine NA subtypes. Each PCR reaction had 10^0^–10^4^ five different concentrations of EID_50_.

Nine NA plasmids ranging from 10^0^ to 10^9^ copies/μl were used to determine the detection limit of the combined multiplex rRT-PCR. Standard curves of detections for each plasmid showed a wide dynamic range and high correlation coefficient, R^2^ > 0.99. Taking Ct = 35 as the cut-off value, the detection limit of the multiplex rRT-PCR was 10–100 copies per reaction (Fig. 3). Nine AIV isolates with different NA subtypes were also used to determine the detection limit of the developed rRT-PCR (Table 5, Fig. 4). The detection limits for N1, N4, N5, N7, and N8 subtypes were 10 EID_50_/PCR, while the detection limits for N2, N3, N6, and N9 subtypes were 100 EID_50_/PCR.

**Table 5:** Detection limits of the single and multiplex assays for 9 NA subtypes.

Group	NA subtype	Fluorescent dye	Limits of detection (EID_50_/PCR)	
			Single assays	Multiplex assays
1	H5N1	FAM	1	10
	H1N4	HEX	10	10
	H5N5	Texas Red	1	10
2	H10N3	FAM	10	100
	H5N2	HEX	10	100
	H4N6	Texas Red	100	100
3	H10N7	FAM	1	10
	H7N9	HEX	10	100
	H3N8	Texas Red	10	10

### Detection of artificial mixed samples

To evaluate whether the developed rRT-PCR could be used to identify the NA subtype in a mixed sample, three concentrations (100 EID_50_, 10 EID_50_ and 1 EID_50_) of each viral NA subtype in three combinations were tested. All nine NA subtypes were detected by the developed rRT-PCR in the equal concentration mixtures of 100 EID_50_ with Ct values from 25.83 to 34.84 (Table 6). Only some NA subtypes were detected in the equal concentration mixtures of 10 EID_50_. All primer-probe pairs reacted only with their corresponding NA subtype, suggesting that the developed rRT-PCR is able to detect different NA subtype from mixed samples.

**Table 6:** Detection of samples with mixed NA subtype viruses using the developed rRT-PCR.

EID_50_	Fluorescent dye	NA subtype	Group		
			A	B	C
100 EID_50_	FAM	N1	27.86^a^	—^b^	—
		N3	—	34.26	—
		N7	—		27.23
	HEX	N2	—	34.84	—
		N4	26.12	—	—
		N9		—	34.52
	Texas Red	N5	28.71	—	—
		N6	—	34.67	—
		N8	—	—	25.83
10 EID_50_	FAM	N1	34.79	—	—
		N3	—	—	—
		N7	—	—	33.90
	HEX	N2	—	—	—
		N4	34.69	—	—
		N9	—	—	—
	Texas Red	N5	33.29	—	—
		N6	—	—	—
		N8	—	—	34.21
1 EID_50_	FAM	N1	—	—	—
		N3	—	—	—
		N7	—	—	—
	HEX	N2	—	—	—
		N4	—	—	—
		N9	—	—	—
	Texas Red	N5	—	—	—
		N6	—	—	—
		N8	—	—	—

^a^Represents Ct value of the sample; ^b^not detected.

### Detection of samples from experimentally infected chickens

**Figure 5:**
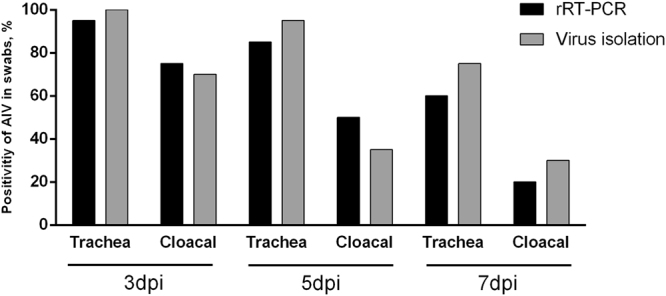
Virus shedding rates in trachea and cloacal swabs of H9N2-infected SPF chickens detected by the multiplex rRT-PCR and virus isolation. Three-week-old SPF White Leghorn chickens were inoculated intranasally with 10^6^ EID_50_ of AIV H9N2 in a 0.2 ml volume (n = 20). Trachea and cloacal swabs were collected from chickens at 3, 5, and 7 dpi, and resuspended in 1 ml PBS for total RNA extraction followed by rRT-PCR and virus isolation.

To compare the sensitivity of the developed rRT-PCR to virus isolation, twenty trachea and cloacal swabs of H9N2-infected SPF chicken were collected at 3, 5, and 7 days post-infection (dpi) for detection of virus shedding. The multiplex rRT-PCR detected viral RNA in 19 trachea and 15 cloacal swabs at 3 dpi, 17 tracheas and 10 cloacal swabs at 5 dpi, and 12 trachea and 4 cloacal swabs at 7 dpi. Viruses were isolated from 20 trachea and 14 cloacal swabs at 3 dpi, 19 trachea and 7 cloacal swabs at 5 dpi, and 15 trachea and 6 cloacal swabs at 7 dpi. These data suggest that the sensitivity of the multiplex rRT-PCR is comparable to virus isolation (Fig. 5).

### Detection of clinical samples

A total of 500 cloacal swab samples were detected by the developed rRT-PCR and the NA subtypes of AIVs were confirmed by virus isolation and sequencing of NA gene (Table 7). The rRT-PCR results showed that 7.4% of poultry (37/500) from two LBMs were positive for AIV, and the positive rate in chickens was the highest (10.3%; 31/300), followed by geese (5.0%; 5/100), pigeons (1.0%; 1/100); no virus RNA was detected in duck samples (0.0%; n = 100). There were five NA subtypes detected by rRT-PCR, including N1, N2, N6, N8, and N9. Of the 35 positive samples, 28 samples contained only one NA subtype, while 7 samples contained two NA subtypes. For single infections, 22/28 samples were confirmed as the expected NA subtype while 6/28 samples failed to be sequenced. For co-infections, 6/7 samples were confirmed as the major NA subtype with the lower Ct value in the multiplex rRT-PCR assay, while 1 sample was not sequenced.

**Table 7:** Results from cloacal swab samples from LBMs using multiplex rRT-PCR and NA gene sequencing.

Isolates	Host				Multiplex rRT-PCR assay							HA subtype	Gene sequencing
		NAsubtype	FAM	HEX					Texas Red				
			N1	N3	N7	N2	N4	N9	N5	N6	N8		
A-1	chicken	N1	33.13^a^	—^b^	—	—	—	—	—	—	—	H5	N1
A-2	chicken	N6/N9	—	—	—	—	—	27.10	—	34.04	—	H7	N9
A-3	chicken	N9	—	—	—	—	—	27.75	—	—	—	H7	N9
A-4	chicken	N9	—	—	—	—	—	23.83	—	—	—	H7	N9
A-5	chicken	N9	—	—	—	—	—	23.02	—	—	—	H7	N9
A-6	chicken	N9	—	—	—	—	—	27.92	—	—	—	H7	N9
A-7	chicken	N9	—	—	—	—	—	28.39	—	—	—	H7	N9
A-8	chicken	N9	—	—	—	—	—	28.61	—	—	—	H7	N9
A-9	chicken	N9	—	—	—	—	—	29.93	—	—	—	H7	N9
A-10	chicken	N9	—	—	—	—	—	30.64	—	—	—	H7	N9
A-11	chicken	N6/N9	—	—	—	—	—	32.76	—	34.64	—	H7	N9
A-12	goose	N2	—	—	—	27.68	—	—	—	—	—	H5/H9	N2
A-13	goose	N2	—	—	—	31.56	—	—	—	—	—	H5	/^c^
A-14	goose	N2	—	—	—	31.48	—	—	—	—	—	H5	/
A-15	goose	N2	—	—	—	25.45	—	—	—	—	—	H5/H9	N2
A-16	goose	N2	—	—	—	32.43	—	—	—	—	—	H5/H9	/
A-17	chicken	N9	—	—	—	—	—	25.31	—	—	—	H7	N9
A-18	chicken	N9	—	—	—	—	—	25.89	—	—	—	H7	N9
A-19	chicken	N9	—	—	—	—	—	32.70	—	—	—	H7	N9
A-20	chicken	N9	—	—	—	—	—	33.33	—	—	—	H7	N9
A-21	chicken	N9	—	—	—	—	—	33.65	—	—	—	H7	N9
A-22	chicken	N9	—	—	—	—	—	33.07	—	—	—	H7	N9
A-23	chicken	N9	—	—	—	—	—	30.92	—	—	—	H7	N9
A-24	chicken	N1	33.73	—	—	—	—	—	—	—	—	H5	/
A-25	chicken	N9	—	—	—	—	—	32.46	—	—	—	H7	N9
B-1	chicken	N9	—	—	—	—	—	26.54	—	—	—	H7	N9
B-2	chicken	N9	—	—	—	—	—	31.56	—	—	—	H7	N9
B-3	chicken	N2/N6	—	—	—	25.14	—	—	—	33.37	—	H5/H9	N2
B-4	chicken	N2/N6	—	—	—	25.11	—	—	—	34.37	—	H5	N2
B-5	chicken	N1	31.93	—	—	—	—	—	—	—	—	H5	N1
B-6	chicken	N1/N8	31.51	—	—	—	—	—	—	—	33.94	H5	N1
B-7	chicken	N6/N9	—	—	—	—	—	31.85	—	34.21	—	H7	N9
B-8	chicken	N2	—	—	—	33.00	—	—	—	—	—	H9	/
B-9	chicken	N2/N6	—	—	—	33.35	—	—	—	28.72	—	H5/H9	/
B-10	chicken	N2	—	—	—	32.78	—	—	—	—	—	H9	/
B-11	pigeon	N2	—	—	—	29.28	—	—	—	—	—	H5	N2
B-12	chicken	N2	—	—	—	28.72	—	—	—	—	—	H5	N2

^a^Represents Ct value of the sample; ^b^not detected; ^c^failed sequencing.

## Discussion

Due to nonspecific clinical signs at the early phase of AIV infections, rapid and accurate identification of different NA subtypes combined with specific HA are necessary to implement disease control measures. Although neuraminidase inhibition (NI) assays have long been used as the Office International Des Epizooties (OIE) standard for NA subtyping, molecular assays based on RT-PCR or real-time RT-PCR have been successfully applied to diagnose some NA types of AIVs^17^. Indeed, multiplex PCR and multiplex fluorescence real-time quantitative PCR techniques are widely used for the detection^21,22^. However, most of these published methods only covered part of the NA types or showed poor sensitivity.

In this study, we developed a combined multiplex probe one-step real-time RT-PCR assay to detect all nine NA types simultaneously and without cross reactivity (Fig. 3). Five common fluorescent probes (FAM, HEX, Cy5, VIC and Texas Red) are often used in detection methods^23–25^. After screening and validating fluorescent dyes and combinations of three NA subtypes, the probes with FAM, HEX, or Texas Red, and the combinations of three specific NA subtypes were set up for this assay. The detection limit of nine NA subtypes was less than 100 copies of cDNA per reaction, similar to multiple PCR-based assays^20^, and superior to SYBR Green-Based Real-Time Reverse Transcription-PCR^26^. When 500 cloacal swab samples were analyzed, the results for the developed rRT-PCR and the reference method (virus isolation and sequencing) were in agreement for 81.1% of the cloacal swab samples (Table 7). AIV coinfections are commonly found in clinical samples, especially in clocal swab samples collected from apparently healthy poultry. We also confirmed that the developed rRT-PCR could detect different NA subtypes in a mixed NA subtype sample. Therefore, the samples with N9/N6 or N2/N6 or N1/N8 double positive by the developed rRT-PCR should be considered as coinfection.

It remains difficult to evaluate the specificity and sensitivity of this method for clinical samples, especially because samples containing the N4, N5, and N7 NA types are limited. We propose that the methods described here could be extended to the routine diagnosis and epidemiological detection of AIV infections.

## Materials and Methods

### Ethical approval

The Jiangsu Administrative Committee for Laboratory Animals approved all animal studies (Permit Number: SYXKSU-2007-0005) according to the guidelines of Jiangsu Laboratory Animal Welfare and Ethical of Jiangsu Administrative Committee of Laboratory Animals.

### Virus strains

Nine AIV isolates of nine NA subtypes were either isolated from Chinese live-bird markets (LBMs) or kindly provided by Professor Jinhua Liu from China Agricultural University (Table 8). Other avian viruses such as Newcastle disease virus (NDV), adenovirus, avian infectious bronchitis virus (IBV), Marek’s disease virus (MDV), avian infectious bursal disease virus (IBDV) were obtained from the Key Laboratory for Animal Infectious Diseases, Ministry of Agriculture, Yangzhou University, Jiangsu, China and used for specificity tests.

**Table 8:** AIV strains used for PCR validation and sensitivity assays.

Avian influenza virus strains	NA subtype	Genbank accession number
A/Duck/Eastern/China/22/005(H5N1)	N1	EU429783
A/Duck/Eastern China/264/02(H5N2)	N2	EU429744
A/Duck/Eastern/China/488/2003(H10N3)	N3	EU429712
A/Duck/Eastern China/01/2005(H8N4)	N4	EU429780
A/Shearwater/Australia/1/1972(H6N5)	N5	EU429794
A/Duck/Eastern/China/01/2007(H4N6)	N6	EU429790
A/Chicken/Germany/N/1949(H10N7)	N7	EU429796
A/Duck/Eastern/China/90/2004(H3N8)	N8	EU429700
A/Chicken/Jiangsu/WJ-14/2015(H7N9)	N9	MF276768

All avian viruses were propagated in the allantoic cavities of 10-day-old embryonated chicken eggs. The median egg infectious dose (EID_50_) of each AIV used in sensitivity tests was determined by inoculating serial 10-fold dilutions of virus into embryonated chicken eggs^27^ and calculated according to the method of Reed and Muench^28^. All live highly pathogenic avian influenza viruses were handled in the authorized animal biosafety level 3 facilities at Yangzhou University.

### Primers and probes design

To design NA-specific primers and probes of the multiple rRT-PCR, 1,084 complete NA genomic sequences combined with different HA subtypes were downloaded from the GenBank database of the National Center for Biotechnology Information (NCBI) (http://www.ncbi.nlm.nih.gov/nuccore/). Multiple alignments of NA sequences were constructed using Clustal W within the MegAlign module of LASERGENE package (DNASTAR Inc., Madison, WI, USA) to identify conserved regions. Primers and probes for each NA subtype were designed using the Primer Premier 5 (version 5.0, Applied Biosystems), and potentials for dimerization, cross-linking, and secondary structures were analyzed using LASERGENE software. The probes were differently labeled with the fluorescent dyes FAM (5-Carboxyfluorescein), HEX (5-hexachloro-fluorescein), or Texas Red.

### RNA purification and cDNA synthesis

Total RNAs were extracted from allantoic fluids, trachea, or cloacal swabs by using the High-Pure PCR Template Preparation Kit (Roche Molecular Biochemicals, Indianapolis, IN, USA) according to the manufacturer’s instructions^29^. Total RNA was eluted in 20 μl elution buffer for each sample. Viral RNA was transcribed to cDNA using a HiScript 1st Stand cDNA Synthesis kit (Vazyme, China), with 45 min of incubation at 50 °C followed by 85 °C for 5 s.

### Preparation of NA plasmids

The cDNA was produced as described above. RT-PCR reaction mixtures (25 μl volume) contained: 8.5 μl nuclease free water, 12.5 μl 2 × Taq Master Mix (Vazyme, China), 2 μl cDNA, and 1 μl (0.4 μM) of each primer. Reaction conditions were: 94 °C for 5 min, 35 cycles of 94 °C for 30 s, 55 °C for 30 s, and 72 °C for 1 min, followed by 72 °C for 7 min at the end of the reaction. PCR products of the expected lengths were purified using a Axygen PCR Purification kit (Corning, USA), and then cloned using pEASY-T3 cloning kit (Transgen, China). Plasmids were extracted by using a TIANprep Mini Plasmid Kit (TIANGEN, China) and identified by using PCR and DNA sequencing.

### Establish the multiple rRT-PCR

To determine whether primer/probe pairs and protocol were suitable for subtyping NA of AIVs, nine PCR reactions for nine NA subtypes were carried out simultaneously in a set of tubes with each pair of the NA-specific primer/probe (Table 1). The rRT-PCR reactions were performed using one-step TaqProbe qRT-PCR kit (ABM, Canada) in reaction mixtures (25 μl volume) containing: 7 μl nuclease free water, 12.5 μl TaqProbe 2 × qRT-PCR Master Mix, 0.5 μl qRT-PCR Enzyme Mix, 2 μl RNA, and 1 μl (0.4 μM) of each primer and probe. The identical thermal profile was adopted to detect the distinct subtypes simultaneously and within the same run. rRT-PCR consisted of one cycle of a 15 min reverse-transcription step at 42 °C, then 95 °C for 10 min, 40 cycles of 95 °C for 15 s, and 60 °C for 1 min. Fluorescence emissions were measured during the annealing-extension step and detection were conducted with the LightCycler Nano system. The threshold cycle number (Ct value) represented the cycle number at which the fluorescence exceeded the threshold. Gel electrophoresis was performed to confirm the size and purity of the products after the rRT-PCR.

Using the developed reaction system, we tested each primer-probe set in the single assay, and then combined them into triplex reactions for multiple rRT-PCR assays.

### Specificity

Nine plasmids containing N1-N9 at 10^5^copies/μl of each plasmid, were combined as templates for specificity tests. AIV isolates (n = 111) and several other avian pathogens were also used to assess the specificity of the developed rRT-PCR. HA subtypes were identified using a standard HI assay with polyclonal chicken antisera^30^. The NA subtypes were determined by sequence analysis^9,31^. Briefly, the NA genes of these viruses were amplified using primers and PCR conditions described by Hoffmann^9^. The PCR products were subcloned into pEASY-T3 vector (Promega, Madison, WI, USA) and sequenced. The NA subtypes were identified by nucleotide BLAST searches of viral nucleotide sequences available from NCBI, Bethesda, MD, USA (http://www.ncbi.nlm.nih.gov/BLAST/).

Extraction of total RNA, the reaction volume and amplification cycles were performed as described above. The result of each reaction was determined by calculating the Ct value.

### Detection limit

Each group of 10-fold serial dilutions of 9 NA plasmids, ranging from 10^0^ to 10^9^ copies/μl, were used as standard preparations to assess the detection limit of viral RNA copy loading. Also, 10-fold serially diluted allantoic fluids containing 10^0^–10^4^ EID_50_ nine NA subtype AIVs were used to prepare viral RNA and cDNA for detection limit of infective virus.

### Detection of mixed samples

Three groups of mixed viruses, including group A: N1, N4, and N5; group B: N2, N3, and N6; group C: N7, N8, and N9, were used as samples for the developed rRT-PCR detection. The equal concentrations of each NA subtype virus were mixed range from 100 EID_50_ to 1 EID_50_ dilution.

### Detecting samples from experimentally infected chickens

Three-week-old specific-pathogen-free (SPF) White Leghorn chickens from Beijing Meiliyaweitong Experimental Animal Technology Co., Ltd, were inoculated intranasally with 10^6^ EID_50_ of AIV H9N2 in a 0.2 ml volume (n = 20). Trachea and cloacal swabs were collected from chickens at 3, 5, and 7 days post-infection (dpi), and resuspended in 1 ml PBS for rRT-PCR detection and virus isolation^29^. For virus isolation, the samples were inoculated into the allantoic cavities of 10-day-old embryonated chicken eggs, after 3 days of incubation at 35 °C, the presence of hemagglutinating agents was determined by performing hemagglutination assays using 1% chicken erythrocytes.

### Evaluation using clinical swab samples

Cloacal swabs (n = 500) were collected from apparently healthy poultry in two LBMs (A and B) of Jiangsu province in China in 2016. The swabs were collected in 1 ml PBS supplemented with antibiotics (penicillin 10,000 unit/mL, streptomycin 10 mg/mL, gentamycin 250 μg/mL, kanamycin, 250 μg/mL) and used for extraction of total RNA followed by rRT-PCR and virus isolation.

## Electronic supplementary material

Supplementary Information
