# Supplementary material for: Development of a multiplex probe combination-based one-step real-time reverse transcription-PCR for NA subtype typing of avian influenza virus
Source: Sci Rep. 2017 Oct 18;7:13455. doi: 10.1038/s41598-017-13768-4 (PMC5647442; doi:10.1038/s41598-017-13768-4)
Supplement: Supplementary file 1 — Supplementary Information [file 41598_2017_13768_MOESM1_ESM.doc]

**Supplementary Information**

**Development of a multiplex probe combination-based one-step real-time reverse transcription-PCR for NA subtype typing of avian influenza virus**

Zhihao Sun^1,2,3ξ^, Tao Qin^1,2,3ξ^, Feifei Meng^1,2,3^, Sujuan Chen^1,2,3^, Daxin Peng^1,2,3^*, Xiufan Liu^1,2,3^


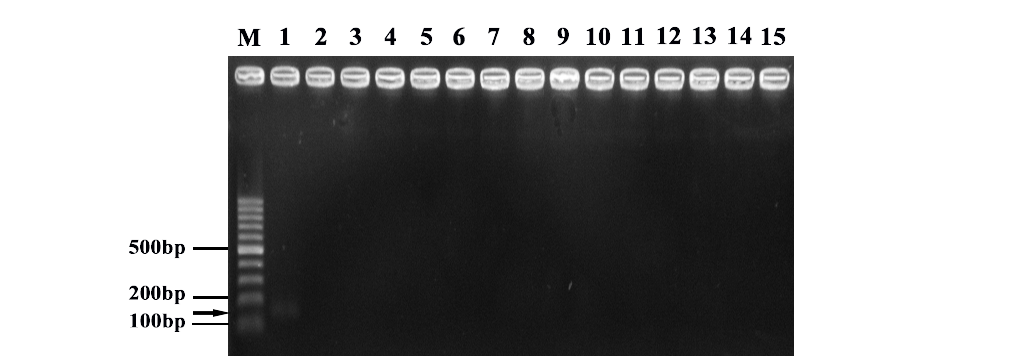


**Supplementary Figure 1-1.** RT-PCR analysis of the subtype-specific primer-probe pairs of N1 with N1-N9 subtype strains and other avian viruses. Lane M: 100 bp ladder molecular size marker; lane 1: N1; lanes 2-9: N2-N9; lane 10: NDV; lane 11: IBV; lane 12: adenovirus; lane 13: MDV; lane 14: IBDV; lane 15: nuclease free water; The arrow points the expected size of PCR product.


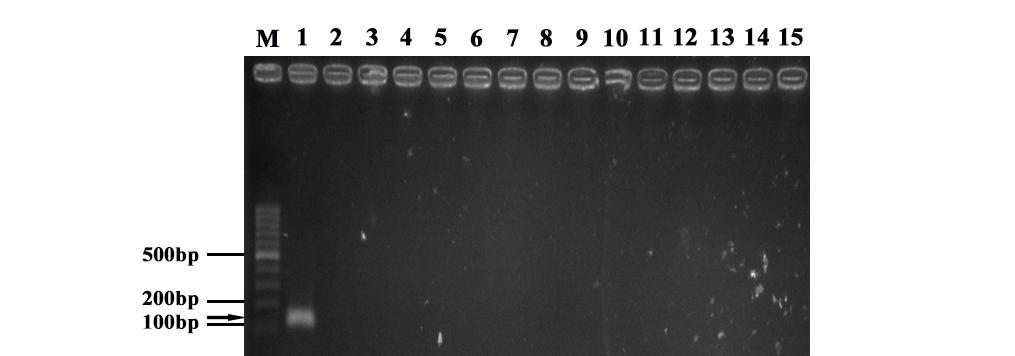


**Supplementary Figure 1-2.** RT-PCR analysis of the subtype-specific primer-probe pairs of N2 with N1-N9 subtype strains and other avian viruses. Lane M: 100 bp ladder molecular size marker; lane 1: N2; lanes 2-9: N1-N9; lane 10: NDV; lane 11: IBV; lane 12: adenovirus; lane 13: MDV; lane 14: IBDV; lane 15: nuclease free water. The arrow points the expected size of PCR product.


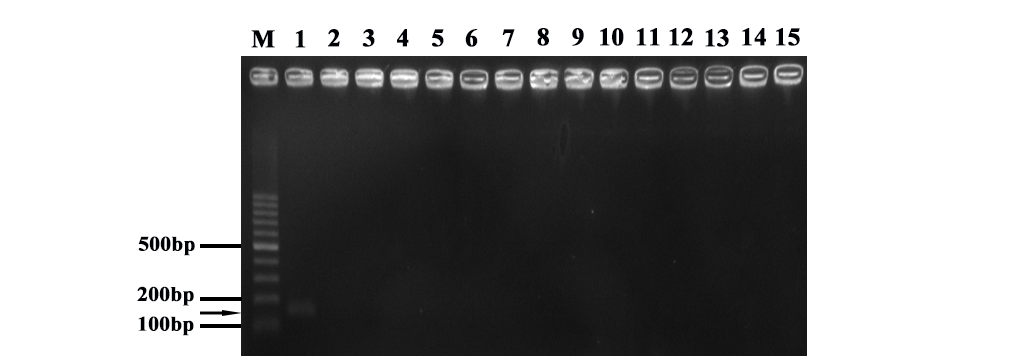


**Supplementary Figure 1-3.** RT-PCR analysis of the subtype-specific primer-probe pairs of N3 with N1-N9 subtype strains and other avian viruses. Lane M: 100 bp ladder molecular size marker; lane 1: N3; lanes 2-9: N1-N9; lane 10: NDV; lane 11: IBV; lane 12: adenovirus; lane 13: MDV; lane 14: IBDV; lane 15: nuclease free water. The arrow points the expected size of PCR product.


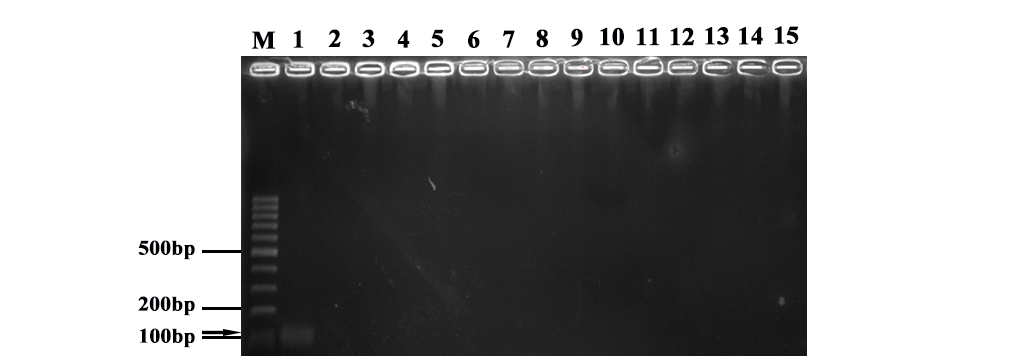


**Supplementary Figure 1-4.** RT-PCR analysis of the subtype-specific primer-probe pairs of N4 with N1-N9 subtype strains and other avian viruses. Lane M: 100 bp ladder molecular size marker; lane 1: N4; lanes 2-9: N1-N9; lane 10: NDV; lane 11: IBV; lane 12: adenovirus; lane 13: MDV; lane 14: IBDV; lane 15: nuclease free water. The arrow points the expected size of PCR product.


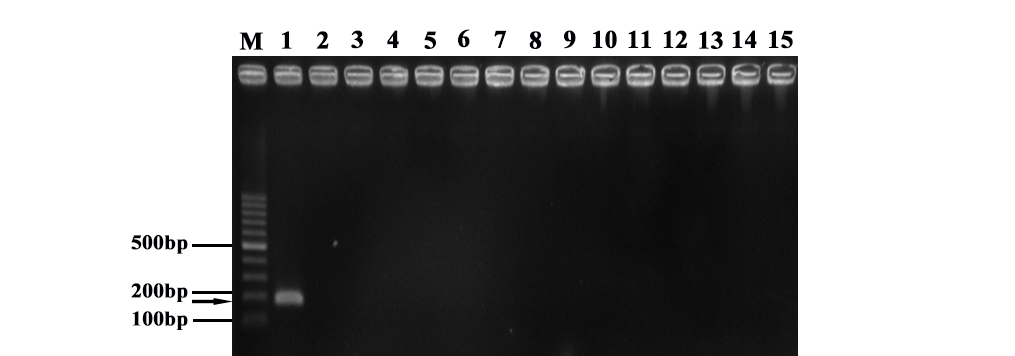


**Supplementary Figure 1-5.** RT-PCR analysis of the subtype-specific primer-probe pairs of N5 with N1-N9 subtype strains and other avian viruses. Lane M: 100 bp ladder molecular size marker; lane 1: N5; lanes 2-9: N1-N9; lane 10: NDV; lane 11: IBV; lane 12: adenovirus; lane 13: MDV; lane 14: IBDV; lane 15: nuclease free water. The arrow points the expected size of PCR product.


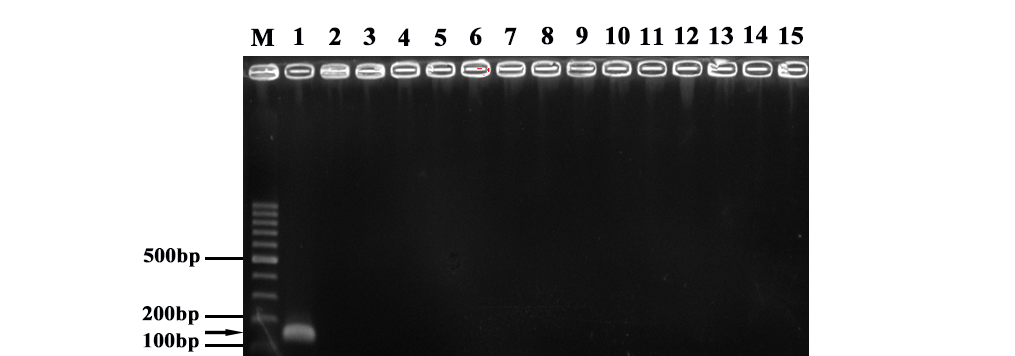


**Supplementary Figure 1-6.** RT-PCR analysis of the subtype-specific primer-probe pairs of N6 with N1-N9 subtype strains and other avian viruses. Lane M: 100 bp ladder molecular size marker; lane 1: N6; lanes 2-9: N1-N9; lane 10: NDV; lane 11: IBV; lane 12: adenovirus; lane 13: MDV; lane 14: IBDV; lane 15: nuclease free water. The arrow points the expected size of PCR product.


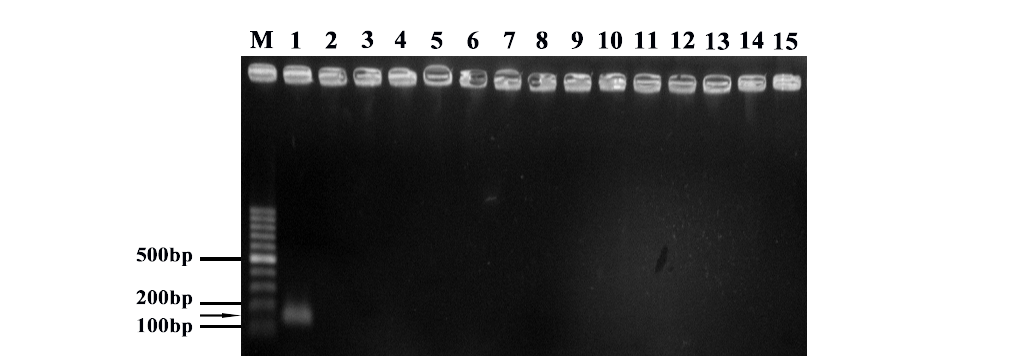


**Supplementary Figure 1-7.** RT-PCR analysis of the subtype-specific primer-probe pairs of N7 with N1-N9 subtype strains and other avian viruses. Lane M: 100 bp ladder molecular size marker; lane 1: N7; lanes 2-9: N1-N9; lane 10: NDV; lane 11: IBV; lane 12: adenovirus; lane 13: MDV; lane 14: IBDV; lane 15: nuclease free water. The arrow points the expected size of PCR product.


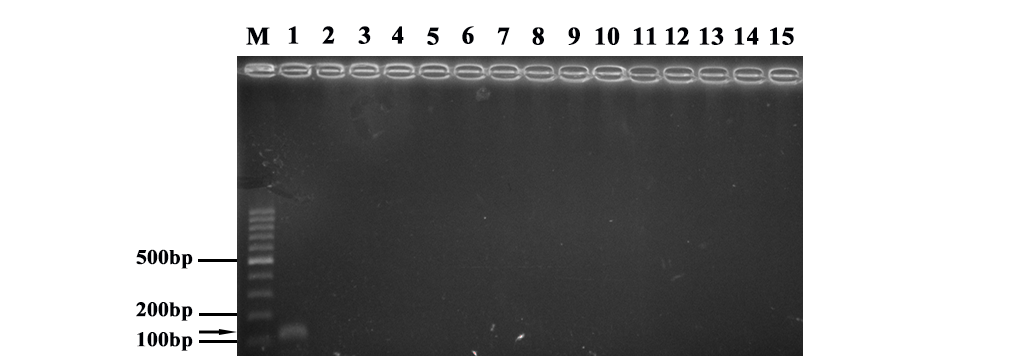


**Supplementary Figure 1-8.** RT-PCR analysis of the subtype-specific primer-probe pairs of N8 with N1-N9 subtype strains and other avian viruses. Lane M: 100 bp ladder molecular size marker; lane 1: N8; lanes 2-9: N1-N9; lane 10: NDV; lane 11: IBV; lane 12: adenovirus; lane 13: MDV; lane 14: IBDV; lane 15: nuclease free water. The arrow points the expected size of PCR product.


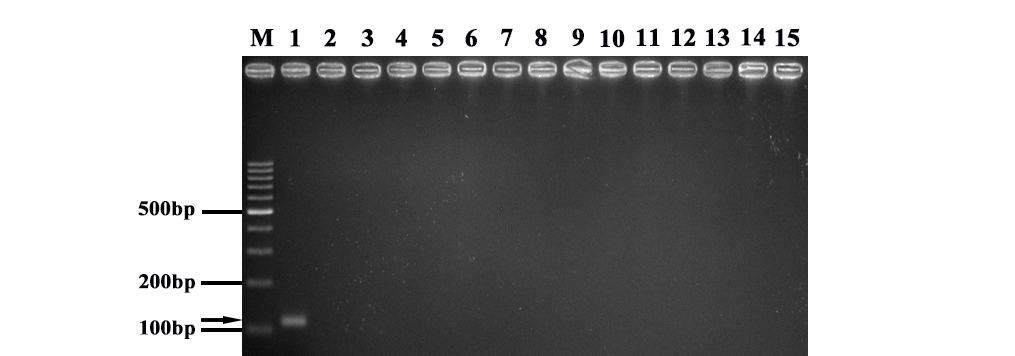


**Supplementary Figure 1-9.** RT-PCR analysis of the subtype-specific primer-probe pairs of N9 with N1-N9 subtype strains and other avian viruses. Lane M: 100 bp ladder molecular size marker; lane 1: N9; lanes 2-9: N1-N8; lane 10: NDV; lane 11: IBV; lane 12: adenovirus; lane 13: MDV; lane 14: IBDV; lane 15: nuclease free water. The arrow points the expected size of PCR product.
